# Endoscopic ultrasound-guided hepaticogastrostomy using a novel double-lumen tapered dilator combined with a 22-G needle

**DOI:** 10.1055/a-2530-3297

**Published:** 2025-02-18

**Authors:** Takeshi Ogura, Kimi Bessho, Takafumi Kanadani, Nobuhiro Hattori, Hiroki Nishikawa

**Affiliations:** 1 38588 Endoscopy Center, Osaka Medical and Pharmaceutical University Hospital, Osaka, Japan; 2 13010 2nd Department of Internal Medicine, Osaka Medical and Pharmaceutical University, Osaka, Japan

Endoscopic ultrasound (EUS)-guided hepaticogastrostomy (HGS) can be indicated for failed endoscopic retrograde cholangiopancreatography (ERCP). Recently, various transendoscopic ultrasonography/endosonography-created route procedures have been developed, such as antegrade bile duct stone removal and stricture management 1 . These procedures may be mainly indicated for benign biliary disease. Compared with malignant biliary disease, EUS-HGS might be challenging in cases of benign biliary disease because the intrahepatic bile duct is not very dilated. Indeed, compared with a meta-analysis of EUS-HGS for malignant biliary disease 2 , the technical success rate might be lower for benign biliary disease 3 . A 22 G needle may improve the technical success of bile duct puncturing. However, because a 0.018-inch guidewire, which has little stiffness, should be used, device insertion into the biliary tract may be challenging. To overcome this, a novel dilation device (Meissa; Japan Lifeline, Tokyo, Japan) has been developed ( Fig. 1 ). This device has a 2.3-Fr tip and a maximum diameter of 7.4 Fr. In addition, a 2-cm side hole is provided from the tip. Contrast medium injection, aspiration of bile juice, and 0.025-inch guidewire insertion can be performed. Therefore, if this device is used, the double-guidewire technique can be performed without additional device exchange with a 0.018-inch guidewire. A challenging case of EUS-HGS due to a non-dilated bile duct is described.

**Fig. 1:**
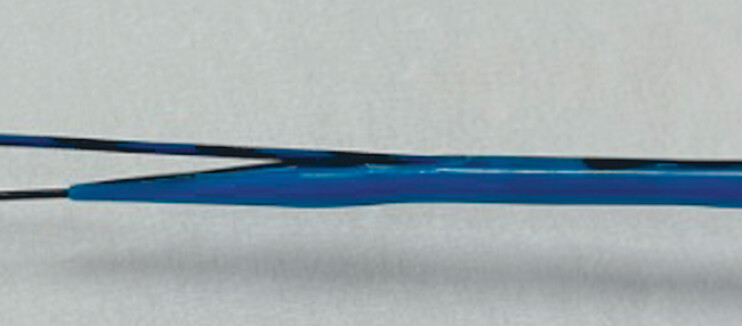
A novel double lumen dilation device.

An 80-year-old man was referred to our hospital due to acute cholangitis caused by a common bile duct stone. He underwent distal gastrectomy with a Roux-en-Y anastomosis. Stone removal was then performed through an enteroscopic approach in another hospital but failed. Therefore, EUS-HGS was attempted. Since the diameter of the intrahepatic bile duct was only 1 mm ( Fig. 2 , arrow), a 22 G needle was selected as the puncture needle. The intrahepatic bile duct was successfully punctured and contrast medium was also injected. Then, a 0.018-inch guidewire was inserted ( Fig. 3 ). Next, insertion of the novel dilation device was attempted, and it was easily inserted into the biliary tract. Subsequently, bile juice was aspirated and contrast medium was injected. On cholangiography, a common bile duct stone was observed. A 0.025-inch guidewire was inserted through the side hole of the novel dilator ( Fig. 4 ). After tract dilation, an 8.5-Fr stent delivery system was easily inserted and successfully deployed from the intrahepatic bile duct to the stomach ( Fig. 5 ) without any adverse events ( Video 1 ). After the cholangitis resolved, antegrade removal of the stone was successfully performed.

**Fig. 2:**
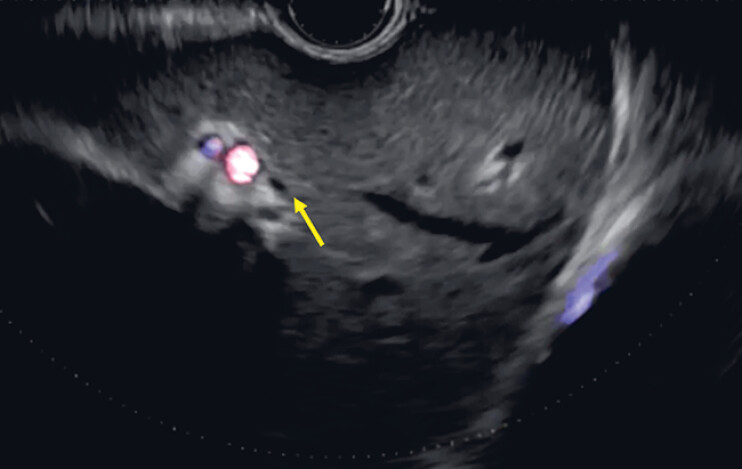
The diameter of the intrahepatic bile duct is only 1 mm (arrow).

**Fig. 3:**
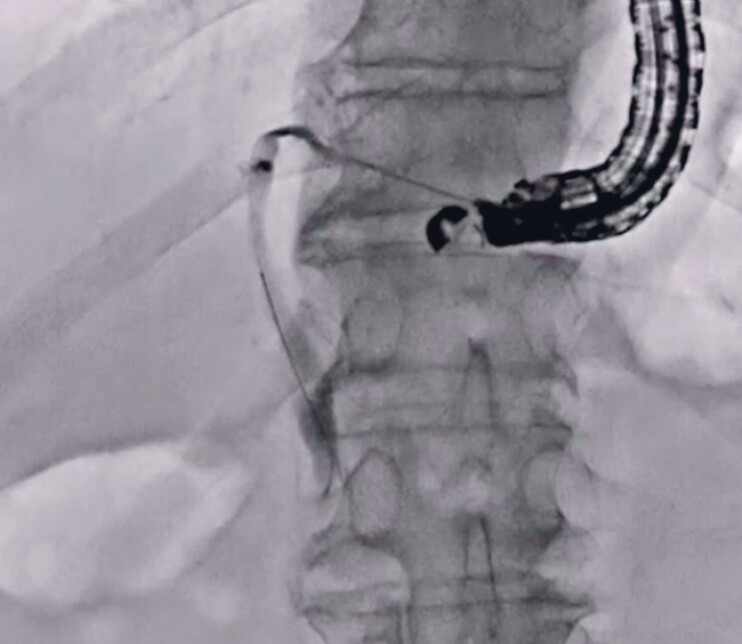
After successful bile duct puncture using 22 G needle, a 0.018-inch guidewire is inserted.

**Fig. 4:**
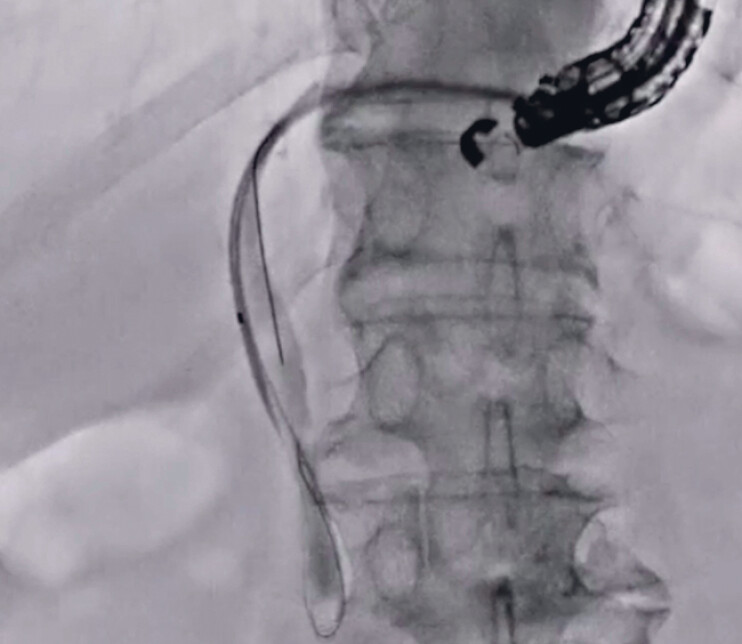
A 0.025-inch guidewire is inserted through the side hole of the novel dilator.

**Fig. 5:**
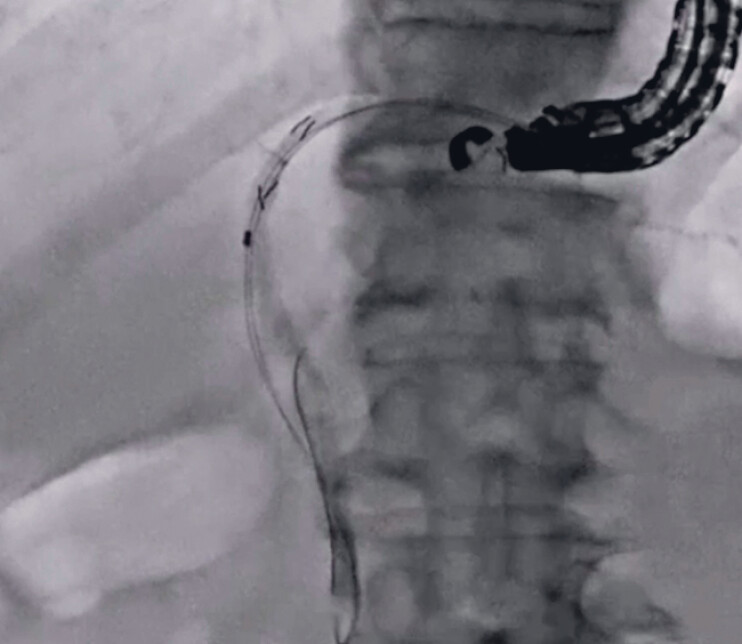
After tract dilation, an 8.5-Fr stent delivery system is easily inserted and successfully deployed from the intrahepatic bile duct to the stomach.

Successful endoscopic ultrasound-guided hepaticogastrostomy using a novel double-lumen tapered dilator combined with a 22 G needle.Video 1

In conclusion, this dilation device may be useful for EUS-HGS using a 22 G needle combined with a 0.018-inch guidewire. Additional cases are needed to further evaluate this device.

Endoscopy_UCTN_Code_TTT_1AS_2AH

## Notes

Endoscopy E-Videos https://eref.thieme.de/e-videos
*E-Videos* is an open access online section of the journal *Endoscopy* , reporting on interesting cases and new techniques in gastroenterological endoscopy. All papers include a high-quality video and are published with a Creative Commons CC-BY license. Endoscopy E-Videos qualify for HINARI discounts and waivers and eligibility is automatically checked during the submission process. We grant 100% waivers to articles whose corresponding authors are based in Group A countries and 50% waivers to those who are based in Group B countries as classified by Research4Life (see: https://www.research4life.org/access/eligibility/ ). This section has its own submission website at https://mc.manuscriptcentral.com/e-videos .
